# S189. CARVING A MORE SPECIFIC SUBTYPE OF SCHIZOPHRENIA FOR GENETIC STUDIES: SPORADIC SCHIZOAFFECTIVE BIPOLAR TYPE

**DOI:** 10.1093/schbul/sby018.976

**Published:** 2018-04-01

**Authors:** Nicolaas van der Merwe, Maria Karayiorgou, René Ehlers, Johannes Roos

**Affiliations:** 1 University of Pretoria; 2 Columbia University New York

## Abstract

**Background:**

Schizophrenia is a heterogeneous group of disorders. The familial-sporadic distinction has been considered under a range of genetic models. Research supports a strong association of de novo copy mutations with sporadic schizophrenia. The aim of the study was to determine a more homogenous phenotype for genetic research via comparison of various clinical and socio-demographic variables in familial and sporadic schizophrenia.

**Methods:**

A cross-sectional observational design was used. This study included 384 participants with schizophrenia/schizoaffective disorder from an Afrikaner founder population in South Africa that previously participated in genetic research. A comprehensive data capturing sheet was completed from a pre-existing database that contains information obtained from the Diagnostic Interview for Genetic Studies, chronological clinical summary reports and additional sources of information. The study protocol was approved by the Research Ethics Committee from the Faculty of Health Sciences at the University of Pretoria. Logit models were fitted using the backward elimination procedure to investigate relationships where the dependent variable was binary. Odds ratio estimates were calculated for independent variables that were significant in the model. The Kruskal-Wallis test was conducted to compare the means of groups. For cases where there were significant differences a post-hoc test with a Bonferroni correction was done to determine which groups differ significantly.

**Results:**

There were 214 familial and 170 sporadic subjects. 279 had a diagnosis of schizophrenia, 66 schizoaffective, bipolar type and 39 schizoaffective disorder, depressive type. 242 were male and 142 female. The age at onset of the primary psychiatric diagnosis, season of birth, co-morbid diagnoses, symptomatology, suicidality history and marital status weren’t significantly different when considering the combined schizophrenia/schizoaffective disorder group and its relationship to familiality. Early deviant behaviour was however decreased in the sporadic group. These findings were replicated when analysing schizophrenia independently from schizoaffective disorder. The sporadic schizoaffective disorder, bipolar type did however have a significantly lower age at onset (mean 20.18 versus 25.07 years), 8.8 times more hallucinations, 6.6 times more odd behaviour before the age of 10 and were 2.8 times more likely to be single. The bipolar type also had 2.9 times more suicide attempts as opposed to ideation. This finding wasn’t statistically significant. The sporadic schizoaffective group overall was 2.2 times more likely to abuse substances. The depressive type didn’t differ significantly with regards to age at onset, season of birth, co-morbidities, early deviant behaviour, symptomatology, suicidality or marital status.

**Discussion:**

The combined schizophrenia/schizoaffective group didn’t differ significantly, nor did schizophrenia and schizoaffective disorder depressive type when analysed independently. The sporadic schizoaffective bipolar type differed significantly on multiple important variables that suggest a poorer prognosis and increased disease severity. The sporadic schizoaffective bipolar type forms a more homogenous group, with genetic studies hinting at relatively specific genetic risk factors. Studies elucidating the genetic architecture of this group could prove invaluable in clarifying the aetiology of schizophrenia.

